# Residual Cardiovascular Risk: Role of Remnants Cholesterol, Monocyte/HDL Ratio and Lipoprotein Ratios on Personalized Cardiovascular Prevention

**DOI:** 10.3390/jpm14050460

**Published:** 2024-04-26

**Authors:** Vincenzo Sucato, Francesco Comparato, Antonella Ortello, Alfredo Ruggero Galassi, Giuseppina Novo

**Affiliations:** Division of Cardiology, Department of Health Promotion, Mother and Child Care, Internal Medicine and Medical Specialties (PROMISE) “G. D’Alessandro”, University Hospital Paolo Giaccone, University of Palermo, 90127 Palermo, Italy; francesco.c1409@gmail.com (F.C.); antonellaortello@gmail.com (A.O.); giuseppina.novo@unipa.it (G.N.)

**Keywords:** residual cardiovascular risk, remnants cholesterol, monocyte/HDL ratio, lipoprotein ratios, cardiovascular prevention

## Abstract

Cardiovascular diseases represent the leading cause of death in the world and are subject to limitations in prevention strategies despite the use of very effective drugs. The concept of residual risk (RR) is intrinsically related to that of global risk of which it represents a very significant percentage. In the cardiovascular field, the term RR refers to the probability of incurring a major cardiovascular event, despite adequate control of the risk factors present in the individual patient. A significant portion of the RR in the cardiovascular field results from the underestimation of additional risk factors not subjected to adequate intervention such as, for example, triglyceride levels in patients treated for the presence of hypertension and/or hypercholesterolemia. The control of the RR therefore appears as an essential condition for the effective reduction of the global risk profile and is based on an integrated intervention that combines all the different prevention strategies derived from the available evidence and capable of interacting on the basis of a strengthening reciprocal between lifestyle and pharmacological and nutraceutical intervention methods.

## 1. Introduction

Residual cardiovascular risk is the probability of developing a major cardiovascular event after placing the patient being treated as recommended by standards of care [1].

Residual cardiovascular risk has multiple components:

(a) The risk associated with known and unknown but non-modifiable factors (e.g., age, sex, ethnicity, genetic predisposition);
(b) The risk associated with factors that are known and modifiable but not completely correct and correctable (HDL and Triglyceride levels, abdominal adiposity, blood pressure, insulin resistance, smoking, etc.).

Dyslipidemia is responsible for approximately 55% of the risk of developing a myocardial infarction. Cardiovascular disease stands as the primary cause of morbidity and mortality globally, with dyslipidemia emerging as a significant risk factor [2]. The primary characteristic of dyslipidemia is the rise in LDL cholesterol levels, which is closely linked to an elevated risk of cardiovascular disease, particularly atherosclerotic cardiovascular disease (ASCVD) [3,4]. Multiple epidemiological, clinical, and experimental investigations have underscored the critical importance of LDL cholesterol, including its oxidized variant, as primary catalysts in the advancement of atherosclerosis [5]. Consequently, reducing LDL cholesterol levels stands as one of the most common approaches in clinical practice for treating and preventing ASCVD [6].

Statin therapy represents therefore the cornerstone of this treatment and the trials clinics in which they were used in primary and secondary prevention; although, they have highlighted that a reduction in the relative risk of cardiovascular events, after treatment, however, leave the patients exposed to a substantial residual risk.

Persistent lipid profile abnormalities despite treatment with statins contribute significantly to residual risk of cardiovascular disease and therefore put the emphasis on the clinical importance of adopting therapeutic strategies that are not limited to reducing LDL cholesterol, but which also act on HDL cholesterol and triglycerides [7].

## 2. Residual Cardiovascular Risk: New Evidence

**Figure 1:**
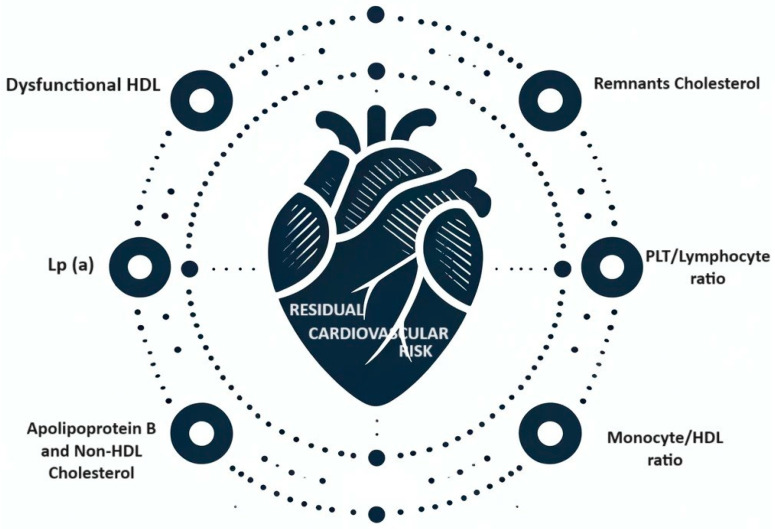
Residual cardiovascular risk.

Traditional risk factors alone cannot fully explain the high impact of coronary events in effectively treated patients; in fact, the classic calculators for estimating cardiovascular risk that only consider conventional risk factors, such as the Framingham risk score, could underestimate the risk in this particular population [8]. For this reason, it is also necessary to individuate new emerging cardiovascular risk factors (Figure 1).

### 2.1. Dysfunctional HDL

Dysfunctional HDL (dys-HDL) are HDL cholesterol particles with high pro-atherogenic characteristics, characterized by the loss of typical antioxidant properties, becoming carriers of oxidized lipids themselves. The early studies emphasized the importance of distinguishing between the loss of HDL function and the acquisition of dysfunction. It was noted that HDL could lose its anti-inflammatory properties and even develop proinflammatory characteristics. However, there are instances where HDL’s ability to deliver cholesterol esters via SCARB1 may be reduced, indicating only a partial loss of function. Subsequent research has shown that the extent of HDL’s normal function loss and dysfunction gain can vary depending on its different biological activities. Some studies have indicated a complete loss of function and dysfunction gain concerning anti-inflammatory, vasodilatory, and antiapoptotic activities. However, when it comes to antioxidative activity and cholesterol efflux capacity, the deficiency observed in HDL’s normal function falls short of complete dysfunction [9].

### 2.2. Lipoprotein (a)

The Lp(a) particle resembles LDL in its cholesterol-rich composition and consists of apolipoprotein B covalently linked to apolipoprotein(a) [apo(a)]. Its connection to cardiovascular risk, atherosclerosis, and coronary artery disease has gained attention [10,11]. Elevated Lp(a) levels have been associated with increased risks of coronary and cerebrovascular events, peripheral artery disease, heart failure, and atrial fibrillation [12]. Studies, such as the Copenhagen City Heart Study involving 9330 individuals, indicate a progressive rise in myocardial infarction risk over a decade with increasing Lp(a) levels [13]. Notably, Lp(a) is associated with premature atherosclerosis and juvenile acute coronary syndrome (ACS), with younger individuals exhibiting a higher probability of ACS with increasing Lp(a) levels compared to middle-aged individuals. Recent categorization of Lp(a) levels suggests that levels below 30 mg/dL (75 nmol/L) indicate a lower cardiovascular risk, while levels between 30 and 50 mg/dL (75–125 nmol/L) represent a “gray zone” in terms of risk [12]. Controversy exists regarding the effects of statins on Lp(a) levels, with some studies suggesting an increase in statin-treated patients, particularly those with small apo(a) phenotypes. However, statins are crucial for patients with elevated Lp(a) levels due to their efficacy in intense LDL reduction [12]. Niacin lowers ApoB-containing particle levels through various mechanisms, and the potential impact of PCSK9 inhibitors on Lp(a) levels alongside niacin therapy has been proposed. Clinical trials have shown a median reduction in Lp(a) percentage with PCSK9 inhibitors, but this reduction does not translate into a decrease in cardiovascular events [14,15]. Combination therapy with niacin and statins poses an increased risk of adverse effects. ASOs targeting ApoB mRNA, such as mipomersen, can reduce both LDL cholesterol and Lp(a) levels, but their side effects limit their use [16]. CETP inhibitors, like anacetrapib, have shown promise in reducing Lp(a) levels, primarily through diminishing apo(a) production [17]. Emerging therapies targeting apo(a) mRNA, such as pelacarsen and olpasiran, demonstrate significant reductions in Lp(a) levels without notable safety concerns. Clinical trials of these therapies show promising results in reducing cardiovascular events. A phase 1 trial of muvalaplin, a novel orally administered drug targeting the non-covalent interaction between apo(a) and apoB, has been recently completed [18]. During ESC Congress 2023, results demonstrated dose-dependent increases in plasma concentrations with muvalaplin, along with decreased Lp(a) levels and no associated safety or tolerability issues.

## 3. Remnants Cholesterol

Several clinical trials demonstrated that in patients with dyslipidemia undergoing cholesterol-lowering treatment, a significant residual cardiovascular risk continues to persist, despite reaching the “target” values for LDL cholesterol.

This appears to be due to the presence of high plasma levels of *triglyceride-rich lipoproteins* (LRT) and remnant cholesterol (RC).

The concentration of LRTs is closely related to triglyceridemia values, and the cardiovascular risk associated with these lipoproteins derives from the fact that they are hydrolysed into RC particles. The latter are lipoproteins rich in cholesterol esters, smaller than LRT and therefore are believed to have an atherogenic potential similar to LDL, in fact the plasma concentration of RC appears to be related to cardiovascular risk independently of LDL cholesterol.

Under fasting conditions, the RCs correspond to the cholesterol content present in very low density lipoproteins (VLDL) and their partially triglyceride depleted remnants (IDL). Conversely, in non-fasting conditions, RCs also include the cholesterol present in the resulting chylomicrons from intestinal absorption of dietary fats. Consequently, RCs correspond to all plasma cholesterol minus HDL cholesterol and LDL cholesterol [19].

After meals, enterocytes produce and release chylomicrons, lipoproteins rich in triglycerides and cholesterol esters introduced through diet into the lymphatic circulation. In the liver, regardless of meals, hepatocytes synthesize and release VLDL, lipoproteins rich in endogenous triglycerides and cholesterol esters into the systemic circulation. Chylomicrons and VLDL constitute triglyceride-rich lipoproteins (LRTs). Lipoprotein lipases (LPL) present in the circulation and tissues hydrolyze the triglycerides of VLDL and chylomicrons transforming them, respectively, into VLDL/IDL remnants and chylomicron remnants. The whole of the cholesterol contained in these lipoproteins rich in triglycerides constitutes the cholesterol remnants. The smaller dimensions of these particles facilitate their penetration into the arterial wall, where they accumulate within the intimal layer. This accumulation initiates an inflammatory cascade, involving the activation of macrophages and platelets, contributing to the progression of the atherosclerotic plaque [20].

The increase in cardiovascular risk determined by high CR values has been highlighted in several studies. In a study conducted by Fukushima et al., out of 240 diabetic patients, half of whom also suffered from coronary heart disease, it was demonstrated that the increase in RC values was an independent risk factor for coronary heart disease. Furthermore, diabetic patients with coronary heart disease showed higher RC values than patients suffering only from diabetes, and following statistical analysis, it was highlighted that higher RC values corresponded to a greater frequency of future acute coronary events [21].

In some studies, conducted over 90,000 individuals from the general Danish population, over a period of 22 years, it was observed that the progressive increase in RC values measured in non-fasting patients was significantly correlated with an increased risk of coronary heart disease and acute coronary events, as well as mortality for all causes. Furthermore, it was highlighted that individuals with RC values > 43 mg/dL had a 2–3 times greater risk of ischemic heart disease compared to subjects with values < 15 mg/dL. In particular, it was found that an increase of 1 mmol/L (39 mg/dL) of RCs in conditions of not fasting was associated with a risk increase of 2.8 times in coronary heart disease [22,23].

Vallejo-Vaz, A.J. et al. observed that RC values, in patients suffering from ischemic heart disease, were significantly correlated with the development of major adverse cardiovascular events (MACEs) [11]. However, the reduction of 1 standard deviation (SD) in RC values following treatment with atorvastatin resulted in a decrease in the incidence of MACE independently of the reduction in LDL cholesterol [24].

Qin, Z. et al. measured the plasma levels of RCs in 2312 patients with type 2 diabetes mellitus undergoing coronary angioplasty and highlighted that patients with higher values of these lipoproteins more frequently suffered in-stent restenosis (ISR). In fact, the statistical analysis showed that the RC values were independently associated with the ISR, and in particular, the baseline value of 0.505 mmol/L (19.5 mg/dL was identified as the optimal cut-off to predict this complication of coronary angioplasty [25].

## 4. Monocyte/HDL Ratio

The monocyte-to-HDL-cholesterol ratio (*MHR*), *calculated by dividing* the monocyte count in peripheral blood by the HDL cholesterol value, is a recent indicator of systemic inflammation and is also considered a predictor of cardiovascular events. Monocytes are involved in the release of pro-inflammatory and pro-oxidative cytokines at sites of inflammation, playing an important role in chronic inflammation and cardiovascular disease. Furthermore, these inflammatory cells are the main cells that infiltrate the arterial wall in the initial stages of atherogenesis. Conversely, HDL cholesterol has anti-inflammatory and antioxidant effects which are expressed through the Inhibition of the production and the mobilization of monocytes, as well as through blocking the migration of macrophages into the arterial wall and the removal of cholesterol molecules from these cells.

Korkmaz, A. et al. measured MHR values in 301 patients undergoing coronary angiography for the evaluation of fractional flow reserve (FFR) of angiographically intermediate stenoses (occlusion between 40% and 70%). It was demonstrated that patients with hemodynamically significant stenosis (FFR ≤ 0.80) had higher MHR values, and through multivariate analysis, this ratio was found to be an independent predictor of low FFR values [26].

In particular, an MHR cut-off equal to 12.1 has been identified as being capable of accurately predicting the presence of hemodynamically significant coronary stenoses [27].

Açıkgöz, S.K. et al. highlighted the usefulness of MHR as an independent predictor of in-hospital and long-term (within 5 years) mortality, as well as MACE, in patients with STEMI. In particular, patients were divided into three tertiles based on increasing MHR values, and it was observed that patients belonging to the third tertile (MHR = 30.1) had a higher risk of mortality and MACE. Furthermore, it is worth adding that none of these clinical outcomes were associated with monocyte count or HDL cholesterol when considered individually [28].

In another study conducted on 1170 patients with STEMI undergoing primary angioplasty, it was demonstrated that the frequency of in-stent thrombosis was 2.2 times higher in patients with higher MHR values. In these patients, MHR was found to be an independent predictor of in-stent thrombosis [29].

Furthermore, elevated MHR values were also observed in patients with ischemic stroke, unlike healthy controls who had normal values. In particular, MHR was found to be an independent predictor of 30-day mortality in patients with stroke (with a cut-off value of 17.5 capable of adequately predicting this mortality) [30].

Finally, MHR values were compared between patients with metabolic syndrome and healthy controls, and it was observed that this ratio was significantly increased in patients compared to controls (13.9 vs. 11.1). The MHR was found to be an independent predictor of the presence of metabolic syndrome and could be used as a new indicator of this clinical condition [31].

### 4.1. Platelet/Lymphocyte Ratio

Platelets indeed play a significant role in the development, destabilization, and rupture of atherosclerotic plaques. Conversely, the lymphocyte count serves as an indicator of physiological stress and exhibits an inverse association with inflammation: a reduced lymphocyte count determines an increase in cardiovascular risk and mortality. The Platelet/Linfocyte ratio (*PLR*, *Platelet to Lymphocyte Ratio*), calculated by dividing the platelet count by the lymphocyte count, is a new prognostic marker that integrates the properties of these two parameters *into* one. It provides information on the pathways of platelet aggregation and inflammation and could be more useful and reliable in predicting cardiovascular risk and coronary damage than platelet or lymphocyte counts considered individually [32].

Ayça et al. evaluated the prognostic value of PLR in 440 patients with STEMI undergoing primary angioplasty. Patients were divided into two groups based on low PLR values (<137) and high values (>137). Patients with high PLR values had a greater incidence of the “*no-reflow*” phenomenon and mortality, as well as higher Syntax score values, indicative of a more severe and complex coronary disease [33].

In their study, Zhou et al. investigated the potential correlation between the platelet-to-lymphocyte ratio (PLR) and the severity of coronary heart disease as well as the clinical outcomes following PCI in a Chinese population diagnosed with STEMI. Their findings indicated that PLR exhibited an independent association with both the severity of coronary heart disease and the occurrence of long-term Major Adverse Cardiovascular Events (MACEs) following PCI [34]. Patients with PLR > 171 had more severe coronary stenosis and a worse prognosis, with a higher incidence of MACE during a 5-year follow-up.

It is clear, therefore, how high PLR values can be considered predictors of severe and complex coronary disease, as well as adverse cardiovascular events.

### 4.2. Lipoprotein Ratios

Lipoprotein ratios are indicators of cardiovascular risk with a predictive power higher than that of the respective lipoproteins considered individually. Evaluating these ratios on a routine basis could provide more accurate information regarding patients’ cardiometabolic risk.

Among these ratios, the *total cholesterol*/*HDL*-*C ratio* (TC/HDL), known as the atherogenic index, and the *LDL*/*HDL*-*C ratio stand out.* The latter seems to have a validity similar to that of the TC/HDL-C ratio, since approximately two thirds of plasma cholesterol is found in LDL, and consequently, total cholesterol and LDL cholesterol are closely correlated [35].

Individuals with high CT/HDL-C or LDL/HDL-C values have a greater cardiovascular risk due to the imbalance between the cholesterol transported by atherogenic lipoproteins and that transported by protective lipoproteins. This may be due to an increase in the pro-atherogenic lipoprotein fraction present in the numerator, to a decrease in the anti-atherosclerotic one to the denominator or to both situations [36].

In a review written by Millan, J. et al., several studies are taken into consideration which demonstrate the superiority of these lipoprotein ratios in predicting coronary events and the severity of coronary heart disease, compared to isolated values of CT, LDL-C and HDL-C. In particular, the cut-off of 5.5 for the CT/HDL-C ratio is associated with a moderate cardiovascular risk, while patients with an LDL/HDL-C ratio > 5 have a risk of coronary events six times greater than those that have a value < 5 [37].

Worthy of mention is *non*-*HDL cholesterol* which includes cholesterol contained in all atherogenic lipoproteins (LDL, Lp(a), VLDL, IDL, chylomicrons and their remnants). This specific cholesterol type has been proposed as a secondary therapeutic target for individuals with elevated triglyceride levels and has also been considered a potential surrogate marker for serum apolipoprotein B concentration. Non-HDL cholesterol values are deemed optimal when they are below 130 mg/dL. Elevated levels beyond this threshold are linked with an increased risk of cardiovascular complications [38]. Starting from non-HDL cholesterol it is possible to calculate the non-*HDL*/*HDL-C cholesterol ratio.* Although few studies have evaluated the validity of this lipoprotein ratio in the prediction of cardiovascular disease, it would appear to have a predictive power similar to that of the CT/HDL-C ratio, which led to the LDL/HDL-C ratio [39].

Finally, a final relationship capable of predicting cardiovascular risk is that between triglycerides and HDL cholesterol (TG/HDL-C) used by Luz, P.L. et al., who measured the values of this ratio in 374 patients at high cardiovascular risk undergoing coronary angiography for suspected coronary heart disease and demonstrated that this index strongly correlates with the extent and severity of coronary damage, a correlation which proved to be higher than the values of individual lipoproteins [40]. Specifically, a TG/HDL ratio > 4 is a strong independent predictor for the development of coronary artery disease and microvascular dysfunction [41].

### 4.3. Apolipoprotein B and Non-HDL Cholesterol

Johannesen et al. conducted a study which revealed that in patients undergoing statin treatment, elevated levels of apolipoprotein B (apoB) and non-HDL cholesterol, rather than LDL cholesterol, are correlated with residual risk of all-cause mortality and myocardial infarction. Their discordance analysis indicated that apoB serves as a more precise marker of all-cause mortality risk in statin-treated patients compared to LDL cholesterol or non-HDL cholesterol. Additionally, apoB was identified as a more accurate marker for the risk of myocardial infarction than LDL cholesterol [42].

## 5. Discussion

The motivation for analyzing CT/HDL-C, LDL/HDL-C, TG/HDL-C and non-HDL/HDL-C lies in the fact that many studies have shown that these ratios are indicators of cardiovascular risk with predictive power higher than that of the respective lipoproteins considered individually. The CT/HDL-C ratio, known as the atherogenic index, and the LDL/HDL-C ratio appear to have overlapping validity, since approximately two-thirds of plasma cholesterol is found in LDL. In a written review by Millan, J. et al., it is clear that CT/HDL-C values ≥ 5.5 and LDL/HDL-C > 5 are associated with an increased risk of coronary events [37].

Regarding the TG/HDL-C ratio, Luz, P.L. et al. demonstrated that this ratio strongly correlates with the extent and severity of coronary damage, a correlation which proved to be higher than the values of the individual lipoproteins, and in particular, a TG/HDL ratio > 4 is a strong independent predictor for the development of coronary heart disease [40].

Regarding cholesterol remnants, some studies have highlighted that the progressive increase in RC values measured that non-fasting was significantly correlated with an increased risk of coronary heart disease and acute coronary events, as well as all-cause mortality. Furthermore, it has been shown that individuals with non-fasting CR values > 43 mg/dL had a 2–3 times greater risk of ischemic heart disease compared to subjects with values < 15 mg/dL [22,23]. It was also observed that RC values, in patients suffering from ischemic heart disease, were significantly correlated to the development of major adverse cardiovascular events (MACE), as well as to an increased risk of in-stent restenosis in patients undergoing coronary angioplasty [24,25].

The monocyte/HDL ratio (MHR) is a recent, easily calculable cardiovascular prognostic indicator that indicates the extent of inflammation and oxidative stress.

Furthermore, several studies in the literature confirm the usefulness of this marker in predicting cardiovascular risk. Korkmaz, A. et al. identified an MHR cut-off of 12.1 capable of accurately predicting the presence of hemodynamically significant coronary stenoses in patients undergoing coronary angiography for the evaluation of a fractional flow reserve [27]. A study conducted by Açıkgöz, S.K. et al. highlighted the utility of monocyte-to-HDL cholesterol ratio (MHR) as an independent predictor for both in-hospital and long-term mortality, as well as MACE, in individuals diagnosed with STEMI [31]. Furthermore, they observed that none of these clinical outcomes were associated with monocyte count or HDL cholesterol when considered individually. Consequently, this ratio would appear to have a higher predictive power than the individual parameters that allow its determination. Furthermore, a peculiarity found is that this relationship was found to be independently correlated to the presence of metabolic syndrome [34].

Regarding the platelet/lymphocyte ratio, Zhou et al. observed Chinese patients with STEMI and platelet/lymphocyte ratio values > 171 [43] (Table 1).

A possible role in residual cardiovascular risk can be played by coronary microvascular dysfunction (CMD), which is a prevalent condition that poses significant challenges for cardiologists due to its disabling nature, often manifesting as persistent angina in affected patients [44]. Among individuals, particularly women, who undergo coronary angiography to assess chest pain and stable angina, a considerable proportion (20–30%) exhibit normal coronary angiograms. This specific manifestation is termed primary microvascular angina (MVA) to differentiate it from other forms of MVA associated with identifiable diseases [45]. In primary MVA, the dysfunction primarily affects the pre-arterioles, with endothelial function impairment being a key feature.

In recent years, considerable scientific efforts have been directed towards characterizing MVA and elucidating its pathological mechanisms. The etiology of MVA likely involves multiple mechanisms, and despite advancements, many aspects of its pathogenesis remain unclear. The relative importance of different risk factors and the novel alterations in endothelial function in MVA are areas of ongoing investigation [46].

## Figures and Tables

**Table 1:** Cut-off on new cardiovascular risk factor.

New Cardiovascular Risk Factor	Cut Off
Lipoprotein A	➢ 50 mg/dL
Remenants Cholesterol	➢ 43 mg/dL
Monocyte/HDL-C Ratio	➢ 17.5
Platelet/Lymphocyte Ratio	➢ 171
LDL/HDL-C ratio	➢ 5
Trigliceryd/HDL-ratio	➢ 4

## Data Availability

Not applicable.
